# LD-Aminopterin in the Canine Homologue of Human Atopic Dermatitis: A Randomized, Controlled Trial Reveals Dosing Factors Affecting Optimal Therapy

**DOI:** 10.1371/journal.pone.0108303

**Published:** 2014-09-25

**Authors:** John A. Zebala, Alan Mundell, Linda Messinger, Craig E. Griffin, Aaron D. Schuler, Stuart J. Kahn

**Affiliations:** 1 Syntrix Biosystems, Inc., Auburn, Washington, United States of America; 2 Animal Dermatology Service, Edmonds, Washington, United States of America; 3 Veterinary Referral Center of Colorado, Englewood, Colorado, United States of America; 4 Animal Dermatology Clinic, San Diego, California, United States of America; Colorado State University, United States of America

## Abstract

**Background:**

Options are limited for patients with atopic dermatitis (AD) who do not respond to topical treatments. Antifolate therapy with systemic methotrexate improves the disease, but is associated with adverse effects. The investigational antifolate LD-aminopterin may offer improved safety. It is not known how antifolate dose and dosing frequency affect efficacy in AD, but a primary mechanism is thought to involve the antifolate-mediated accumulation of 5-aminoimidazole-4-carboxamide ribonucleotide (AICAR). However, recent *in vitro* studies indicate that AICAR increases then decreases as a function of antifolate concentration. To address this issue and understand how dosing affects antifolate efficacy in AD, we examined the efficacy and safety of different oral doses and schedules of LD-aminopterin in the canine model of AD.

**Methods and Findings:**

This was a multi-center, double-blind trial involving 75 subjects with canine AD randomized to receive up to 12 weeks of placebo, once-weekly (0.007, 0.014, 0.021 mg/kg) or twice-weekly (0.007 mg/kg) LD-aminopterin. The primary efficacy outcome was the Global Score (GS), a composite of validated measures of disease severity and itch. GS improved in all once-weekly cohorts, with 0.014 mg/kg being optimal and significant (43%, *P*<0.01). The majority of improvement was seen by 8 weeks. In contrast, GS in the twice-weekly cohort was similar to placebo and worse than all once-weekly cohorts. Adverse events were similar across all treated cohorts and placebo.

**Conclusions:**

Once-weekly LD-aminopterin was safe and efficacious in canine AD. Twice-weekly dosing negated efficacy despite having the same daily and weekly dose as effective once-weekly regimens. Optimal dosing in this homologue of human AD correlated with the concentration-selective accumulation of AICAR *in vitro*, consistent with AICAR mediating LD-aminopterin efficacy in AD.

## Introduction

Atopic dermatitis (AD) affects approximately 3% to 5% of the adult population in the western world, and 30% of the worldwide pediatric population [1]. It is a complex, relapsing disease arising from interactions between genes and the environment and is characterized by pruritus, disruption of the epidermal barrier, and IgE-mediated sensitization to food and environmental allergens [2]. The pathogenesis of AD may involve an aberrant Th2 adaptive immune response to innocuous environmental antigens, skin barrier abnormalities, and an inadequate host response to cutaneous microbes [3].

Patients with AD who fail to respond to topical corticosteroids or topical calcineurin inhibitors may require second-line systemic immunosuppressive therapy [4]. Systemic treatment options include cyclosporine, corticosteroids, azathioprine and methotrexate [5], [6]. Cyclosporine and prednisolone are appropriate as short-term treatments [5], the former being nephrotoxic and the latter predisposing to osteoporosis, hypertension and other side-effects [7]. Cyclosporine is also almost entirely metabolized by the liver cytochrome P450 IIIA system, and clinically significant sustained drug-drug interactions can occur during long-term therapy [8]. Caution in the use of azathioprine has been highlighted as well [5], given the heightened risk for hepatosplenic T-cell lymphoma, a rare but frequently lethal form of lymphoma [9]. Despite its well-established record of safety and efficacy, methotrexate is not well tolerated in many patients [10]. The limitations of current systemic treatments have prompted the search for improved treatments that might expand the armamentarium of therapeutic options for patients with AD.

**Figure 1:**
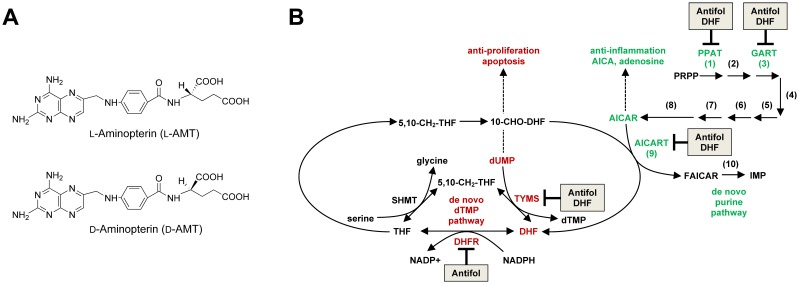
LD-Aminopterin composition and mechanistic model in anti-inflammation. (A) Chemical structure of L-aminopterin (*top*) and D-aminopterin (*bottom*). (B) The anti-inflammatory activity of L-aminopterin and methotrexate have been attributed to inhibition of thymidylate (*red*) and purine (*green*) *de novo* biosynthesis. In the *de novo* pathway of thymidylate (dTMP) synthesis, serine hydroxymethyltransferase (SHMT) catalyzes the conversion of serine and tetrahydrofolate polyglutamates (THF) to 5,10-CH_2_-THF and glycine. Thymidylate synthase (TYMS) converts 5,10-CH_2_-THF and deoxyuridine monophosphate (dUMP) to dihydrofolate polyglutamates (DHF) and dTMP. Dihydrofolate reductase (DHFR) completes the cycle by catalyzing the conversion of DHF to THF in an NADPH-dependent reaction. The purine, inosine monophosphate (IMP), is synthesized *de novo* in 10 chemical steps (shown numbered) catalyzed by six enzymes. The six enzymes are phosphoribosylpyrophosphate amidotransferase (PPAT; 1); a trifunctional enzyme composed of glycinamide ribonucleotide synthetase (GARS; 2), GAR formyltransferase (GART; 3) and aminoimidazole ribonucleotide synthetase (AIRS; 5); formylglycinamidine ribonucleotide synthase (FGAMS; 4); a bifunctional enzyme composed of carboxyaminoimidazole ribonucleotide synthase (CAIRS; 6) and succinoaminoimidazolecarboxamide ribonucleotide synthetase (SAICARS; 7); adenylosuccinate lyase (ASL; 8); and a bifunctional enzyme composed of aminoimidazolecarboxamide ribonucleotide transformylase (AICART; 9) and inosine monophosphate cyclohydrolase (IMPCH; 10). Evidence indicates that 10-formyl-7,8-dihydrofolate (10-CHO-DHF) is the predominant *in vivo* substrate for AICART, making AICART and TYMS the only enzymes to produce the DHFR substrate DHF [69]. Inside the cell, L-aminopterin and methotrexate and their polyglutamate metabolites (antifol) bind with high affinity to DHFR, resulting in accumulation of DHF and depletion of the reduced folate pool. Depletion of folates, as well as the direct inhibition by antifol and DHF, have all been implicated in the inhibition of PPAT, GART, AICART and TYMS [22], [33], [54], [70]. In the case of AICART, the accumulation of DHF may cause this reaction to run backwards, since AICAR is normally driven towards the biosynthesis of FAICAR and IMP by the DHFR-catalyzed reduction of DHF to THF, as the equilibrium of this step actually lies in the direction of AICAR formation [60].

LD-Aminopterin (Syntrix Biosystems, Auburn, WA) is the L- and D-enantiomer of N-[4-[[(2,4-diamino-6-pterdinyl)methyl]amino]benzoyl]-glutamic acid (Figure 1A) [11]. The L-enantiomer is an antifolate congener of methotrexate that is stereoselectively absorbed from LD-aminopterin by the intestinal proton coupled folate transporter [12]. Preclinical and clinical studies indicate it may provide improvements on methotrexate, including better bioavailability [13], [14], greater cell uptake and conversion to active polyglutamylated metabolites [13], [15], less central nervous system toxicity [16], [17], [18], [19], [20], and less liver toxicity [13]. Unlike cyclosporine, LD-aminopterin is not metabolized by human liver microsomes, and thus drug-drug interactions at the cytochrome P450 system are unlikely [12].

Methotrexate, L-aminopterin, and their polyglutamylated metabolites inhibit dihydrofolate reductase and enzymes involved in *de novo* purine and thymidylate synthesis (Figure 1B) [21], [22]. Proposed anti-inflammatory mechanisms have centered on inhibition of *de novo* thymidylate synthesis [23], [24], [25], and inhibition of aminoimidazolecarboxamide ribonucleotide transformylase (AICART), an enzyme involved in *de novo* purine synthesis [26], [27], [28]. Inhibition of *de novo* thymidylate synthesis prevents cell-cycle progression of activated T-cells and induces their apoptosis by a Fas-independent pathway [23], [24], [25], an effect reproduced by several groups [29], [30], [31], [32]. Inhibition of AICART causes increased levels of its substrate, 5-aminoimidazole-4-carboxamide-1-β-D-ribofuranosyl 5′-monophosphate (AICAR), which together with its dephosphorylated metabolite 5-aminoimidazole-4-carboxamide-1-β-D-ribofuranoside (AICA), inhibit AMP deaminase and adenosine deaminase [33], [34], effects that cause an increase in extracellular adenosine [26]. Extracellular adenosine binds adenosine receptors to affect a reduction in inflammation [35]. AICA is also cytotoxic to T lymphocytes, potentiates the cytotoxicity of methotrexate added to cultured T lymphocytes [34], [36], [37] and activates AMP-activated kinase [38], [39].

Funk *et al*. recently demonstrated AICAR increased 115-fold following exposure of an erythroblastoid cell line to 10 nM methotrexate, but decreased with increasing methotrexate concentrations, declining to baseline with 1000 nM methotrexate [40]. In contrast, the substrate for thymidylate synthase, 2′-deoxyuridine 5′-monophosphate (dUMP), displayed concentration-dependent accumulation over the same range of methotrexate concentration. It was suggested that if clinical response is dependent on the accumulation of AICAR, that these *in vitro* findings might predict a clinical therapeutic response paradoxically related to dose.

Initial trials of methotrexate in AD simply adopted the dose and regimen commonly used to treat psoriasis and rheumatoid arthritis [41], [42]. However, given the different underlying pathologic mechanisms between AD and these other autoimmune diseases, it is not clear that the same dosing strategy would be equally applicable. In fact, no study has examined how dose and regimen affect antifolate efficacy in AD, and thus how to best administer antifolate therapy in AD remains a significant unresolved question.

Although mouse models of AD have many practical benefits in the laboratory, they also have significant limitations in how clinically similar their disease is to human AD. In contrast, dogs naturally and commonly develop a pruritic dermatitis that is clinically and immunologically extremely similar to human AD [43]. Like human AD, canine AD is associated with severe pruritus, skin xerosis and increased transepidermal water loss, face and skin fold involvement, spongiotic dermatitis, skin-infiltrating eosinophils, skin infiltration by IgE(+) and CD1c(+) dendritic cells, Th2-dominated immune responses, positive atopy patch test, and IgE-specific responses. Owing to the remarkable similarity with the human disease, it has been suggested that canine AD can not only help answer mechanistic questions related to disease pathogenesis, but also serve as a model for testing of drugs with clinical potential in humans [43].

Here we report the efficacy and safety results from a 12-week dose-ranging randomized, double-blind, placebo-controlled, multi-center trial that tested the efficacy and safety of orally administered LD-aminopterin given once- or twice-weekly to subjects with canine AD. The objective was to examine how efficacy and safety of antifolate therapy varies as a function of dose and schedule. This study provides insights into how to administer antifolate therapy in canine AD that has implications for treating the human disease with LD-aminopterin based on a mechanism aimed at maximizing AICAR accumulation.

## Materials and Methods

### Ethics statement

The study was conducted in compliance with the Veterinary International Committee for Harmonization guidance for good clinical practice and was overseen and approved by a local Institutional Animal Care and Use Committee (North Carolina State University) and a centralized Institutional Animal Care and Use Committee (Infectious Disease Research Institute). Owners of subjects provided written consent for subjects to participate in the study and could withdraw from the study at any time.

### Study design

#### Blinded trial

**Figure 2:**
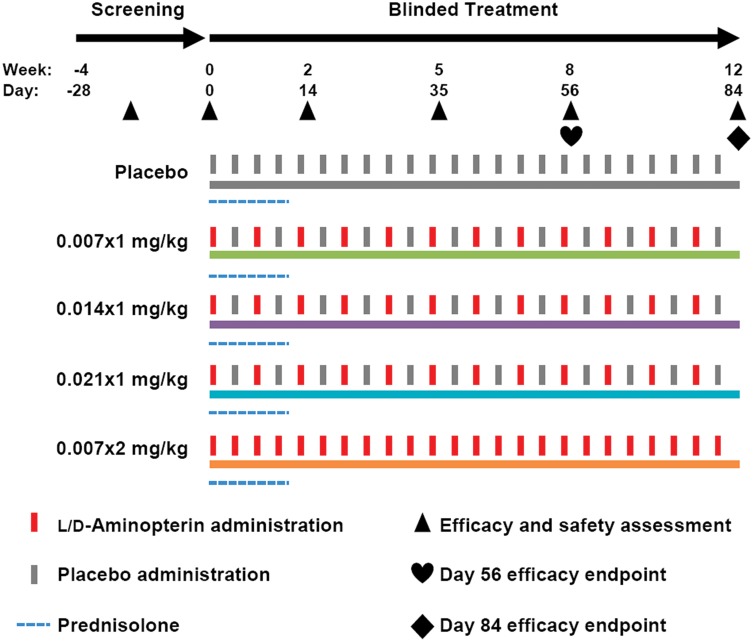
Study flow chart. Randomized subjects with AD were orally administered placebo, or LD-aminopterin once-weekly (0.007×1 mg/kg, 0.014×1 mg/kg, 0.021×1 mg/kg) or twice-weekly (0.007×2 mg/kg).

The study was performed as a double-blinded, randomized, placebo-controlled, parallel-group study conducted at four referral-based specialty practices located in the United States (California, Colorado, North Carolina and Washington) (Figure 2).

Subjects were randomized in a 1∶1∶1∶1∶1 ratio to receive oral doses of placebo, or LD-aminopterin once-weekly (0.007, 0.014 or 0.021 mg/kg) or twice-weekly (0.007×2 mg/kg). Doses are for the free acid of the L-enantiomer. Study drug consisted of either a gelatin capsule containing microcrystalline cellulose (placebo), or a gelatin capsule containing 0.25 mg LD-aminopterin tablets in an appropriate number of whole and/or half tablets to provide the desired dose per subject weight, and backfilled with microcrystalline cellulose. Owners were not required to take any special handling precautions of study drug.

A pre-planned interim efficacy checkpoint at day 56 was instituted based on pilot trial data that indicated responsive subjects achieved the majority of benefit by 4–8 weeks, whereas unresponsive subjects failed to improve with further treatment [44]. Subjects achieving at least 25% GS improvement passed the checkpoint and continued to receive treatment up to day 84. Subjects unable to meet the minimum GS response exited to avoid further futile treatment; their day 56 evaluation became their efficacy endpoint. Efficacy endpoints were therefore from day 56 or 84 per protocol.

Each arm employed a twice-weekly dosing using dummy doses to keep the blind, where the second weekly dose was given 3 days after the first. See below for details on randomization, blinding and dosing compliance. Daily prednisolone (0.5 mg/kg) was offered for the first 14 days without taper to maintain enrollment due to the delayed onset of LD-aminopterin action [44]. No folic acid supplementation was specified. Disease activity was assessed at days 0, 14, 35, 56 and 84.

#### Open-label extension

Subjects from the blinded trial were optionally able to continue on LD-aminopterin in an open-label extension lasting up to 104 weeks. Subjects received other treatments within the standard of care at the discretion of the clinician. Dosing was 0.007–0.021 mg/kg once-weekly at the clinician’s discretion.

### Study population

Inclusion criteria were (i) a diagnosis of canine AD [45], [46]; (ii) moderate-to-severe disease defined by a CADESI score ≥60 and <500 [47]; (iii) age >6 months; (iv) weight 7 to 50 kg; (v) testing to rule out food allergy, flea bite hypersensitivity and external parasites; (vi) absence of fleas and use of a long acting flea adulticide; and (vii) intradermal skin testing or allergen-specific IgE determination confirming the presence of immediate or late-phase hypersensitivity reactions, or reagin immunoglobulins to environmental allergens such as house dust or storage mites, pollens or molds.

Subjects were excluded for (i) pregnancy or lactation; (ii) malignant neoplasia; (iii) diet augmented with fatty acid supplements if the diet was not continued throughout trial; (iv) treatment with long-acting corticosteroids within 6 weeks, oral corticosteroids or cyclosporine within 3 weeks, or oral anti-histamines within 1 week of enrollment; (v) use of anti-allergenic or antipruritic shampoos or conditioners, topical corticosteroids, tacrolimus or cyclosporine within 1 week of enrollment; and (vi) allergen-specific immunotherapy started or changed within 6 months of enrollment, or if the allergen-specific immunotherapy was changed during the study. Antibiotics were permitted per protocol to treat skin infections at the discretion of investigators.

### Assessments

#### Blinded trial

Disease activity was assessed using validated disease measures: PVAS to measure itch [48] and the CADESI to measure disease severity [47]. PVAS yields a possible score from 0 to 10, and CADESI yields a possible score from 0 to 1,240. CADESI and PVAS were assessed at study days 0 (baseline), 14, 35, 56 and 84 (i.e. end of weeks 2, 5, 8 and 12). GS is a composite score that is the product of CADESI and PVAS and thus captures the proportional change in CADESI and PVAS, where GS =  (CADESI×PVAS)/100.

Safety assessments were performed at study days 0, 14, 35, 56 and 84 and consisted of recording all AEs and serious AEs and noting their severity and relationship to study drug. They included the regular monitoring of hematology, blood chemistry, and urine and physical examination. A central laboratory (Antech Diagnostic GLP, Morrisville, NC) was used for analysis of all specimens collected and listed below. Hemoglobin, hematocrit, red blood cell (RBC) count, white blood cell (WBC) count with differential (neutrophils including bands, lymphocytes, monocytes, eosinophils, and basophils), and platelet count were measured at all scheduled study visits within the visit window. Serum chemistries including blood urea nitrogen (BUN), creatinine,, alanine transaminase/serum glutamic pyruvate transaminase (ALT/SGPT), alkaline phosphatase, lactate dehydrogenase (LDH), total protein, and albumin, were measured at all scheduled study visits within the visit window. Urinalysis for specific gravity, protein, glucose, blood, ketones, bilirubin and urobilinogen were performed at scheduled visits on day 0 and 84, or day 56 for subjects who exited the study at the interim efficacy checkpoint.

#### Open-label extension

Safety assessments were every 3 months in the first year and every 6 months in the second year using the same assessments as in the blinded trial.

### Study endpoints

Per protocol, the primary efficacy endpoint was the change in baseline GS at study day 56 or 84. The primary study outcome was to assess the efficacy of four LD-aminopterin dosages in subjects with moderate-to-severe canine AD with respect to the primary efficacy endpoint, and determine the most (or least) effective dosage.

Secondary efficacy endpoints evaluated at study day 56 or 84 were the change in baseline CADESI and PVAS. Secondary outcomes included assessing the efficacy of four LD-aminopterin dosages in subjects with moderate-to-severe canine atopic dermatitis with respect to secondary efficacy endpoints, and determine the most (or least) effective dosage; the effect of LD-aminopterin on each secondary efficacy endpoint over time; the safety of LD-aminopterin by clinical and laboratory AEs as a function of dosage and time.

### Randomization, blinding and dosing compliance

Randomization was performed centrally by Syntrix Biosystems Drug Supply Management. Subjects were randomized 1∶1∶1∶1∶1 into five treatment arms in blocks of five. Randomized blocks were generated using GraphPad QuickCalcs online software (www.graphpad.com/quickcalcs, GraphPad Software, Inc., La Jolla, CA). At randomization, each subject was assigned an identification number that was linked to a treatment arm and a sequentially numbered bottle of blinded study drug. Subject owners did not have contact with one another. All weekly study drug doses were provided in a single similar appearing capsule filled with microcrystalline cellulose. Dosing instructions specified only clear liquids for two hours before taking capsules, except for a small quantity of food to assist in administration. Weight-band-dosing tables were stratified by 1.0 kg increments. To preserve the blind, each arm maintained a schedule of twice-weekly dosing using a dummy dose in the once-weekly treatment schedules, and two dummy doses in the placebo cohort. Dosing compliance was determined by site monitoring and drug accountability (assigned capsules returned). Subject owners, investigator staff, and persons performing the assessments, were blinded to the identity of the treatment.

### Statistical analyses

The sample size calculation was based on assessing four dosages of LD-aminopterin and placebo to determine the most or least effective dosage with respect to the primary efficacy endpoint using Hsu’s multiple comparisons with the best (Hsu’s MCB) test [49]. Assuming a minimum clinically meaningful change in GS of 1.5 (Dr. Thierry Olivry of North Carolina State University), and mean baseline GS of 5.5 and standard deviation of 0.8, both obtained from pilot trial data in subjects (n = 6) with moderate disease [44], a sample size of 15 subjects per cohort was required to achieve a power of 0.9.

The full analysis set consisted of all subjects who were randomized, using the initial randomized dosage, whether the subject ultimately dropped out of the trial or had their dose reduced per protocol. Subjects with missing day 56 or day 84 data were analyzed by the last observation carried forward. Balance in baseline characteristics between cohorts was analyzed by chi-square test for categorical data and one-way ANOVA for continuous data.

The primary outcome, change in baseline GS (absolute and percent change), was analyzed in each cohort by repeated-measures ANOVA. The two-sided type I error was adjusted for multiple cohort comparisons using the Bonferroni correction. The most effective dosage was analyzed using Hsu’s MCB [49]. Hsu’s MCB compares each cohort mean and the “best” of all the other cohort means to identify the best dosage, or reject a dosage as the best dosage. Hsu’s MCB provides joint simultaneous confidence intervals for the differences between the mean baseline change of a dosage cohort minus the maximum of the mean baseline change in each of the other cohorts. If a cohort mean is significantly separated above all other cohort means, it is regarded as ‘the best’ (i.e., lower confidence limit >0). If a cohort mean has at least one cohort mean significantly separated above it, it is rejected as the best dosage (i.e., upper confidence limit <0). Secondary outcomes for CADESI and PVAS were analyzed as above.

*Post hoc* testing was by t-test and Mann-Whitney tests, and categorical data on concomitant medications were analyzed by chi-square test with significance claimed at α = 0.05. Analyses and sample size calculations were performed with commercial software (PASS and NCSS, NCSS, LLC, Kaysville, UT; and GraphPad Prism version 6.00 for Windows, GraphPad Software, La Jolla, CA).

The safety set included all subjects who took at least one dose of study drug and had at least one post-baseline assessment. AEs were summarized by absolute and relative frequencies stratified by cohort and duration treated.

## Results

### Subject baseline characteristics and disposition in the study

**Figure 3:**
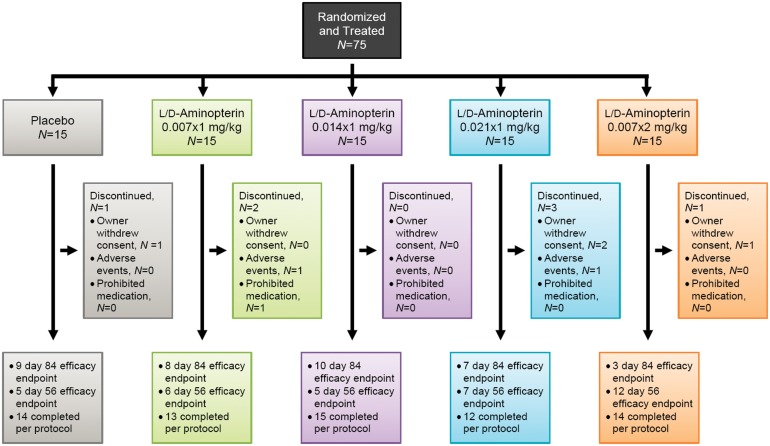
Disposition of subjects. A total of 68 subjects (91%) completed the study per protocol. Discontinuations (9%) were for withdrawal of owner consent (*N* = 2), owner perceived AE (*N* = 2), and prohibited medication (*N* = 1).

Treatment cohorts were balanced with respect to demographic features and baseline disease characteristics (Table 1). The average disease activity in each cohort was severe, defined by a CADESI≥120 [50]. The total population was balanced between moderate (*N* = 36) and severe (*N* = 39) disease. A total of 75 subjects were randomly assigned to receive oral LD-aminopterin or placebo (Figure 3). Four study sites enrolled 5 to 44 subjects each. A total of 68 subjects (90.7%) completed the study per protocol, with 37 subjects treated for 12 weeks and 31 subjects treated up to the interim 8 week efficacy checkpoint. Seven subjects (9.3%) discontinued the study. Drug accountability indicated that 95% (*N* = 71) of all subjects had taken 90% or more of the assigned doses, and this percentage was similar across cohorts.

**Table 1:** Subject demographics and baseline AD characteristics.

		LD-Aminopterin				
	Placebo	0.007×1 mg/kg	0.014×1 mg/kg	0.021×1 mg/kg	0.007×2 mg/kg	
Variable	*N* = 15	*N* = 15	*N* = 15	*N* = 15	*N* = 15	*P*-value^a^
Age, y	6.7±3.5	4.9±2.5	6.8±3.7	6.0±2.6	5.9±3.0	0.48
Male, *N* (%)	9 (60.0)	10 (66.6)	10 (66.6)	10 (66.6)	8 (53.3)	0.91
Body weight, kg	28.7±10.1	23.3±12.2	22.7±12.0	26.1±14.3	17.1±10.9	0.11
GS	11.3±8.5	11.4±4.7	12.3±9.0	10.0±5.9	11.9±7.6	0.93
CADESI	160±105	170±64	159±94	130±56	173±99	0.66
PVAS	6.5±1.5	6.7±1.2	7.4±1.5	7.5±1.5	6.6±1.4	0.19
Nonseasonal, *N* (%)	15 (100.0)	15 (100.0)	14 (93.3)	15 (100.0)	14 (93.3)	0.54

Abbreviations: GS, Global Score; CADESI, Canine Atopic Dermatitis Extent and Severity Index 03; PVAS, Pruritus Visual Analogue Scale.

Data are mean ± SD for continuous variables.

a *P*-values were calculated by chi-square test for categorical data and one-way ANOVA for continuous data.

### Administration of weekly oral LD-aminopterin is efficacious in canine AD

**Figure 4:**
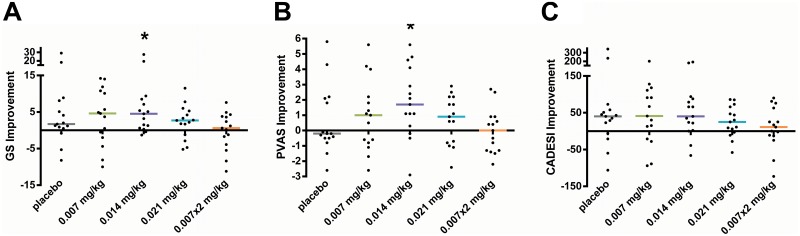
Effect of placebo and LD-aminopterin on canine AD disease measures. Subjects (*N* = 75) with AD were randomized equally to receive placebo, or LD-aminopterin once-weekly (0.007, 0.014 or 0.021 mg/kg) or twice-weekly (0.007×2 mg/kg). Improvement in baseline disease measures were determined for (A) GS, (B) PVAS and (C) CADESI (see Materials and Methods). GS and PVAS improved significantly in the 0.014 mg/kg cohort. *P<0.05. Horizontal bars are medians. Abbreviations: GS, Global Score; PVAS, Pruritus Visual Analogue Scale; CADESI, Canine Atopic Dermatitis Extent and Severity Index 03.

The Global Score (GS) improved significantly in the 0.014×1 mg/kg cohort (Figure 4A). The GS improved by a mean (±SD) of 6.1±7.6 points (95% CI, 1.9–10.3), decreasing from 12.3±9.0 at baseline to 6.2±4.8 after treatment (*P*<0.05). The mean (±SD) percent reduction in baseline GS in the 0.014×1×mg/kg cohort was 43.2±38.0% (95% CI, 22–64%; *P*<0.01).

Treatment with LD-aminopterin also resulted in a significant reduction (*P*<0.05) in itch in the 0.014×1 mg/kg cohort (Figure 4B). The Pruritus Visual Analogue Scale (PVAS) improved by a mean (±SD) of 1.9±2.3 points (95% CI, 0.6–3.2), decreasing from 7.4±1.5 at baseline to 5.5±2.5 after treatment. The mean percent reduction in PVAS in the 0.014×1 mg/kg cohort was 26% (95% CI, 7–43%). Pruritus in 4 of 15 subjects (27%) in the cohort responded with a robust reduction in baseline PVAS≥4 (mean [percent] reduction = 4.8 [65%]).

The change in baseline Canine Atopic Dermatitis Extent and Severity Index 03 (CADESI) was not significant in any cohort, although the 0.014×1 and 0.021×1 mg/kg cohorts had mean (±SD) changes (53±71 and 26±42, respectively) that were significant before adjusting the type I error for multiple comparisons (Figure 4C). There was improvement in mean (±SD) CADESI in the placebo cohort (52±109), but it was not significant even prior to adjusting the type I error for multiple comparisons.

Antibiotics were permitted per protocol to treat skin infections at the discretion of investigators. The mean (±SE) duration of antibiotic treatment was 6.2+3.7 weeks. Antibiotic use was not a confounding factor in the significant efficacy responses to LD-aminopterin in the 0.014×1 mg/kg cohort because antibiotic use was similar across all treatment cohorts and placebo in each consecutive four week treatment period, except in the 0.014×1 mg/kg cohort, where it was lower (Table 2).

**Table 2:** Concomitant medications.

		LD-Aminopterin				
	Placebo	0.007×1 mg/kg	0.014×1 mg/kg	0.021×1 mg/kg	0.007×2 mg/kg	
Medication	*N* = 15	*N* = 15	*N* = 15	*N* = 15	*N* = 15	*P*-value^a^
**Prednisolone,** *N* (%)^b^						
Yes	13 (86.6)	13 (86.6)	13 (86.6)	12 (80.0)	11 (73.3)	
No	2 (13.3)	2 (13.3)	2 (13.3)	3 (20.0)	4 (26.6)	0.83
**Antibiotics,** *N* (%)						
Weeks 0–4	11 (73.3)	12 (80.0)	3 (20.0)	12 (80.0)	12 (80.0)	0.001
Weeks 5–8	7 (46.7)	7 (46.7)	3 (20.0)	6 (40.0)	8 (53.3)	0.397
Weeks 9–12	6 (40.0)	6 (40.0)	3 (20.0)	5 (33.3)	5 (33.3)	0.772
**Prohibited,** *N* (%)						
Yes	0 (0.0)	1 (6.7)	0 (0.0)	0 (0.0)	0 (0.0)	
No	15 (100.0)	14 (93.3)	15 (100.0)	15 (100.0)	15 (100.0)	0.40

a *P*-values calculated by chi-square test.

b During first 14 days.

### Dosing frequency determines optimal efficacy in canine AD

In addition to examining how varying LD-aminopterin dose impacted efficacy in canine AD, this study also examined how the schedule or frequency of administration affected efficacy. Interestingly, all endpoints for twice-weekly LD-aminopterin were no better than placebo, and worse than all once-weekly schedules (Figure 4). CADESI in the twice-weekly regimen was notable for being clearly worse than placebo, though not significantly. The 0.007×2 mg/kg cohort was statistically rejected as the best dosage based on GS and PVAS; each endpoint mean was smaller than, and significantly separated from the corresponding endpoint mean in the 0.014×1 mg/kg cohort (*P*<0.05, Hsu’s MCB).

### A *post hoc* comparison with two weeks of daily prednisolone suggests LD-aminopterin may be highly effective in a subpopulation of canine AD

**Figure 5:**
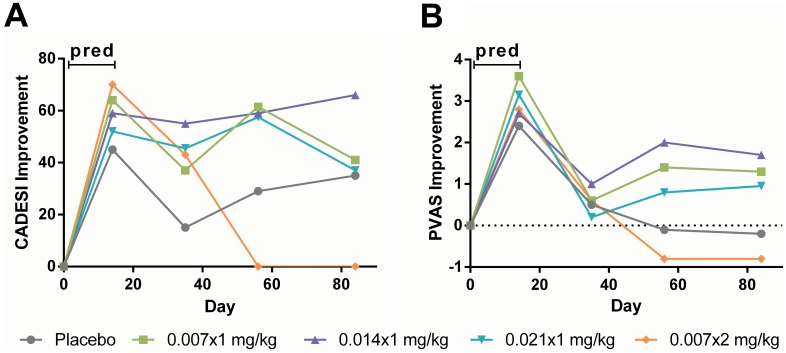
Change in CADESI and PVAS as a function of time in subjects treated with prednisolone and either placebo or LD-aminopterin. Subjects treated with prednisolone (pred) in the first 14 days (*N* = 62) were treated with either placebo (*N* = 13), or LD-aminopterin once-weekly (0.007×1 mg/kg, *N* = 13; 0.014×1 mg/kg, *N* = 13; 0.021×1 mg/kg, *N* = 12) or twice-weekly (0.007×2 mg/kg, *N* = 11). Median improvement in baseline (A) CADESI and (B) PVAS was determined at days 14, 35, 56 and 84. Abbreviations: CADESI, Canine Atopic Dermatitis Extent and Severity Index 03; PVAS, Pruritus Visual Analogue Scale; pred, prednisolone.

Per protocol, subjects were optionally treated with prednisolone in the first 14 days (see Materials and Methods). Subjects treated with prednisolone constituted 83% (*N* = 62), and were distributed similarly across cohorts (Table 2). Two independent time-response profiles were clearly evident for CADESI and PVAS in this sub-population, consistent with prednisolone and LD-aminopterin having distinctly different onsets of action (Figure 5). Whereas the action of LD-aminopterin on PVAS required 56 to 84 days to come to full prominence, prednisolone caused a rapid improvement in PVAS by day 14 that was lost by the time of the primary efficacy endpoint for LD-aminopterin.

The median (mean±SD) improvement in PVAS at day 14 in the prednisolone-treated population (*N* = 62) was 2.8 (2.9±2.4) points, a treatment effect that was notably consistent among all cohorts (Figure 5). In contrast, the median (mean±SD) improvement in PVAS at day 14 in the population not treated with prednisolone (*N* = 13) was 0.0 (0.05±0.6) points. The improvement in PVAS at day 14 in the populations treated and not treated with prednisolone were significantly different (*P*<0.0001 for median and mean). Prednisolone treatment thus served not only to maintain enrollment during the onset of LD-aminopterin efficacy, it also provided an internal positive efficacy control that confirmed the reliability and reproducibility of blinded owner-assessed itch using PVAS.

The median PVAS improvement in the 0.014×1 mg/kg cohort (*N* = 15) due to LD-aminopterin was 61% of the median day 14 PVAS improvement due to prednisolone in the total prednisolone-treated population (*N* = 62). However, this difference was not significant (*P* = 0.22). Of the 62 prednisolone-treated subjects, 21 (33.9%) had robust improvement in PVAS≥4 points at day 14. Among the 0.007×1 mg/kg and 0.014×1 mg/kg cohorts, 7 of 30 subjects (23.3%, all with nonseasonal disease) responded at day 84 with improvement in PVAS≥4 points. The fraction of subjects with improvement in PVAS≥4 after LD-aminopterin was not significantly different than after prednisolone (*P* = 0.34). Of the 7 subjects with improvement in PVAS≥4 after LD-aminopterin, 6 were treated with prednisolone, and had a mean improvement due to prednisolone substantially the same as that seen for the larger (*N* = 62) prednisolone-treated population (2.7±2.1 versus 2.9±2.4, respectively). In these 6 subjects, the mean (±SD) improvement in PVAS due to LD-aminopterin was significantly (77%) greater than from prednisolone (4.8±0.7 versus 2.7±2.1, *P*<0.05).

### LD-Aminopterin is safe and well-tolerated in canine AD

#### Blinded trial

There was no relationship between clinical (Table 3) or laboratory (Table 4) adverse events (AEs), and either dose or schedule. The incidence of AEs in LD-aminopterin treated cohorts was similar to placebo. The most frequently reported AEs (≥5% of 61 total) across all cohorts were gastrointestinal in nature (45.9% [*N* = 28]): diarrhea (26.2% [*N* = 16]) and anorexia (6.6% [*N* = 4]). All were mild in intensity and self-limiting. Abnormalities in liver function as measured by elevations in serum alanine transaminase were most common in placebo, and in all cases were mild and transient (Table 4). The incidence of AEs decreased as a function of time (Table 5). There were no serious AEs, or AEs that led investigators to discontinue study drug, reduce dose, or deviate from protocol.

**Table 3:** Summary of clinical AEs by cohort^a^.

		LD-Aminopterin			
	Placebo	0.007×1 mg/kg	0.014×1 mg/kg	0.021×1 mg/kg	0.007×2 mg/kg
Preferred Term	*N* = 15	*N* = 15	*N* = 15	*N* = 15	*N* = 15
**Subjects with any AE(s)**	**10 (66.6)**	**8 (53.3)**	**7 (46.7)**	**10 (66.6)**	**5 (33.3)**
Death	0	0	0	0	0
Serious AEs	0	0	0	0	0
AE led to discontinuation	0	1 (6.7)^b^	0	1 (6.7)^b^	0
**All AEs in any cohort**	**11 (73.3)**	**12 (80.0)**	**13 (86.7)**	**17 (113.3)**	**8 (53.3)**
Fatigue	1 (6.7)	1 (6.7)	1 (6.7)	2 (13.3)	0
Weight loss	0	0	0	1 (6.7)	0
Diarrhea	2 (13.3)	4 (26.7)	3 (20.0)	5 (33.3)	2 (13.3)
Anorexia	0	1 (6.7)	1 (6.7)	1 (6.7)	1 (6.7)
Vomiting	0	0	1 (6.7)	1 (6.7)	0
Constipation	0	0	1 (6.7)	0	0
Stool increased	1 (6.7)	0	0	0	0
Stool dark color	1 (6.7)	0	0	0	0
Thirst increased	0	2 (13.3)	0	0	0
Halitosis	0	0	1 (6.7)	0	0
Keratoconjunctivitis sicca	0	0	0	1 (6.7)	0
Eye discharge	0	0	0	0	1 (6.7)
Demodicosis^c^	0	1 (6.7)	0	0	1 (6.7)
Pyotraumatic dermatitis	0	0	0	0	1 (6.7)
Skin infection	1 (6.7)	2 (13.3)	2 (13.3)	2 (13.3)	1 (6.7)
Otitis externa	1 (6.7)	0	0	1 (6.7)	1 (6.7)
Urinary incontinence	1 (6.7)	0	1 (6.7)	1 (6.7)	0
Aural hematoma	0	1 (6.7)	0	0	0
Epistaxis	0	0	1 (6.7)	0	0
Anxiety	0	0	0	0	1 (6.7)
Irritability	1 (6.7)	0	0	0	0
Stomach pain	0	0	0	1 (6.7)	0
Dermatitis	1 (6.7)	0	0	0	0
Urticaria	1 (6.7)	0	0	0	0
Tail dysfunction	0	0	0	1 (6.7)	0

Abbreviations: AE, adverse event.

a Expressed as *n* and percent of total subjects in each cohort.

b AE led to discontinuation by subject owner, not by investigator.

c 0.007×1 mg/kg cohort: *Demodex canis* at day 44 post 0.5 mg/kg prednisolone on days 0 to 14; and 0.007×2 mg/kg cohort: *Demodex injai* at day 56 post 1.0 mg/kg prednisolone on days 0 to 14. Demodicosis cleared after one dose of milbemycin oxime, and each subject treated with LD-aminopterin for 24 (0.007×1 mg/kg cohort) and 9 (0.007×2 mg/kg cohort) months in the open-label segment without recurrence.

**Table 4:** Summary of laboratory AEs by cohort^a^.

		LD-Aminopterin			
	Placebo	0.007×1 mg/kg	0.014×1 mg/kg	0.021×1 mg/kg	0.007×2 mg/kg
Laboratory Abnormality	*N* = 15	*N* = 15	*N* = 15	*N* = 15	*N* = 15
Hematocrit Decreased	0	1 (1.3)	1 (1.3)	2 (2.7)	1 (1.3)
RBC Count Decreased	0	0	2 (2.7)	1 (1.3)	0
Thrombocytopenia	1 (1.3)	0	0	0	1 (1.3)
Thrombocytosis	6 (8.0)	7 (9.3)	7 (9.3)	9 (12.0)	4 (5.3)
Leukopenia	1 (1.3)	0	0	0	0
Lymphopenia	2 (2.7)	0	0	1 (1.3)	0
Neutropenia	0	0	1 (1.3)	0	0
Eosinophilia	0	1 (1.3)	0	0	0
BUN Increased	4 (5.3)	4 (5.3)	3 (4.0)	2 (2.7)	2 (2.7)
Creatinine Increased	0	3 (4.0)	1 (1.3)	0	1 (1.3)
Alkaline Phosphatase Increased	6 (8.0)	5 (6.7)	7 (9.3)	6 (8.0)	7 (9.3)
ALT Increased	5 (6.7)	2 (2.7)	3 (4.0)	1 (1.3)	1 (1.3)
Serum Protein Decreased	0	1 (1.3)	0	1 (1.3)	0
Serum Albumin Decreased	2 (2.7)	3 (4.0)	2 (2.7)	1 (1.3)	1 (1.3)
**Total**	**27 (36.0)**	**27 (36.0)**	**27 (36.0)**	**24 (32.0)**	**18 (24.0)**

Abbreviations: RBC, red blood cell; BUN, blood urea nitrogen; ALT, alanine transaminase.

a Expressed as *N* and percent of 75 total subjects.

**Table 5:** Summary of clinical AEs as a function of 4-week intervals^a^.

Preferred Term	0 to 4 Weeks	5 to 8 Weeks	9 to 12 Weeks
**All Categories**	33 (54.1)	21 (34.4)	7 (11.5)
**Constitutional**	3 (4.9)	2 (3.3)	1 (1.6)
Fatigue	3 (4.9)	2 (3.3)	0
Weight loss	0	0	1 (1.6)
**Gastrointestinal**	17 (27.9)	7 (11.5)	4 (6.6)
Diarrhea	10 (16.4)	3 (4.9)	3 (4.9)
Anorexia	2 (3.3)	1 (1.6)	1 (1.6)
Vomiting	1 (1.6)	1 (1.6)	0
ConstipationN	1 (1.6)	0	0
Stool increased	1 (1.6)	0	0
Stool dark color	0	1 (1.6)	0
Thirst increased	2 (3.3)	0	0
Halitosis	0	1 (1.6)	0
**Ocular**	1 (1.6)	1 (1.6)	0
Keratoconjunctivitis sicca	0	1 (1.6)	0
Eye discharge	1 (1.6)	0	0
**Infection**	3 (4.9)	9 (14.8)	2 (3.3)
Demodicosis	0	2 (3.3)	0
Pyotraumatic dermatitis	1 (1.6)	0	0
Skin infection	2 (3.3)	5 (8.2)	1 (1.6)
Otitis externa	0	2 (3.3)	1 (1.6)
**Renal/Genitourinary**	3 (4.9)	0	0
Urinary incontinence	3 (4.9)	0	0
**Hemorrhage**	2 (3.3)	0	0
Aural hematoma	1 (1.6)	0	0
Epistaxis	1 (1.6)	0	0
**Neurology**	2 (3.3)	0	0
Anxiety	1 (1.6)	0	0
Irritability	1 (1.6)	0	0
**Pain**	1 (1.6)	0	0
Stomach pain	1 (1.6)	0	0
**Allergy**	0	1 (1.6)	0
Dermatitis	0	1 (1.6)	0
**Dermatology**	0	1 (1.6)	0
Urticaria	0	1 (1.6)	0
**Musculoskeletal**	1 (1.6)	0	0
Tail dysfunction	1 (1.6)	0	0

a Expressed as *N* and percent of 61 total AEs.

#### Open-label extension

Of the 75 subjects enrolled in the blinded trial, 62 (83%) enrolled in the open-label extension. The doses used in the open-label extension were 0.007 mg/kg (19%), 0.014 mg/kg (57%) and 0.021 mg/kg (24%). Including the 12 weeks of treatment in the blinded trial, 40 (65%) and 23 (37%) subjects were treated for more than 57 and 84 weeks, respectively. The drug was well-tolerated during chronic therapy. There were no clinical serious adverse events or deaths. No clinically significant laboratory adverse events occurred, and there was no dose-dependent trend in the incidence of adverse events for any laboratory test (Table S1). There was no laboratory adverse events that required discontinuation of study drug.

## Discussion

This placebo-controlled study examined how dose and schedule of the investigational antifolate LD-aminopterin affected efficacy and safety in canine AD. Oral LD-aminopterin 0.014 mg/kg given once weekly resulted in efficacy in moderate-to-severe canine AD after 8–12 weeks of treatment, causing a significant reduction in GS and PVAS. An exploratory analysis identified ∼25% of subjects who were highly responsive to the anti-pruritic effect of LD-aminopterin, and enjoyed a significantly larger mean reduction in itch (65%) than from two weeks of daily prednisolone (4.8 versus 2.7 point reduction, or 77% greater). CADESI was also significantly reduced, but only before correcting for multiple comparisons. CADESI was reduced in placebo but not significantly, an effect likely due to permitted antimicrobials [51], and/or carry-over effects of prednisolone used in the first 14 days per protocol [52].

Surprisingly, all efficacy endpoints for twice-weekly 0.007 mg/kg LD-aminopterin were no better than placebo, and worse than all once-weekly schedules. This held whether the once-weekly schedule provided the same daily (0.007 mg/kg) or total weekly (0.014 mg/kg) dose. Based on CADESI, twice-weekly dosing was even worse than placebo, though not significantly. These findings were unexpected and suggest that the schedule of antifolate administration is critical, with a minimum interval between dosings required for efficacy in canine AD.

Like methotrexate, the L-enantiomer of LD-aminopterin potently inhibits dihydrofolate reductase (Figure 1B) [17], [53], which results in the rapid accumulation of dihydrofolate polyglutamates that may reach 20% (∼2 *µ*M) of total intracellular folates from an initial undetectable level [21]. Dihydrofolate polyglutamates at these concentrations are capable of inhibiting the first committed step of purine biosynthesis catalyzed by PPAT and the two transformylase reactions catalyzed by GART and AICART [22]. In addition to dihydrofolate polyglutamates, methotrexate polyglutamates have also been implicated as effectors of inhibition of these three steps of *de novo* purine synthesis [22], [33], [34], [54]. Although AICART inhibition and the accumulation of AICAR and its metabolite AICA have been proposed to mediate anti-inflammatory effects [26], [27], [28], [34], [36], [37], [39], *in vitro* studies with leukemia cells and primary human T lymphocytes indicate that PPAT is the primary site of inhibition of purine biosynthesis by methotrexate [22], [55]. In particular, levels of 5-phosphoribosyl-1-pyrophosphate, the natural PPAT substrate, increase 5-10-fold from 3 to 12 hours in cells exposed in culture to methotrexate at a concentration (0.1 µM) obtained in the plasma of patients undergoing therapy for inflammation, before decreasing to control levels after 24 hours [56], [57]. Thus, methotrexate inhibits PPAT, GART and AICART, but empirically induces AICAR accumulation in patients [58], [59]. Accumulated AICAR may therefore be derived from either selective inhibition of AICART at low antifolate concentrations [40], or from the pools of intermediates that exist between GART and AICART if both enzymes are inhibited non-selectively [22]. In the latter case, the abundance of intermediates may vary from patient to patient, potentially accounting in part for the variability in antifolate clinical efficacy. Another possibility is that AICAR is derived from FAICAR if the accumulation of dihydrofolate polyglutamates causes the AICART reaction to run backward, as suggested by the fact that the equilibrium of this reaction actually lies in the direction of AICAR formation [60].

Persistent inhibition of PPAT, GART and AICART in subjects would be expected to abrogate the downstream accumulation of AICAR and its AICA metabolite, since each would be eliminated from the body without precursors available for the synthesis of additional AICAR [61]. In patients given a single standard anti-inflammatory dose of methotrexate, Smolenska *et al*. demonstrated rapid inhibition of *de novo* purine biosynthesis that was sustained for at least 24–48 hours but that fully reversed by one week after dosing, kinetics that suggest twice-weekly dosing may lead to persistent inhibition of *de novo* purine synthesis [62]. If the anti-inflammatory effect of LD-aminopterin in AD is due to AICAR, an optimal schedule of therapy would require sufficient time between drug pulses to allow enzymes to cycle between states of complete and incomplete inhibition in order to regenerate intermediates in *de novo* purine synthesis and maintain optimally elevated and efficacious levels of AICAR. This mechanism could explain why twice-weekly dosing in this study negated efficacy in AD, despite having the same daily and weekly dose as effective once-weekly regimens.

Support for this model comes from recent *in vitro* studies carried out by Funk *et al*., who demonstrated a 115-fold increase in AICAR following exposure of an erythroblastoid cell line to 10 nM MTX, but subsequently decreased with increasing MTX concentrations, declining to baseline levels with 1000 nM MTX [40]. In contrast, dUMP displayed concentration-dependent accumulation. These observations led these investigators to predict clinical anti-inflammatory responses due to AICAR might be paradoxically related to antifolate dose, whereas a dose-proportional response would be seen if due to inhibition of thymidylate synthase. Toxicity is observed in all subjects administered a sufficiently high dose of LD-aminopterin or methotrexate [12], [14], consistent with the proposal that antifolate toxicity is mediated by thymidylate synthase inhibition [40]. In contrast, the dose-response data for efficacy in this study mirrors the *in vitro* concentration-response findings for AICAR described by Funk *et al*. [40], suggesting that LD-aminopterin efficacy in AD is mediated by AICAR accumulation.

Clinical evidence supportive of this model in humans comes from Radmanesh and colleagues, who observed greater efficacy in psoriatics treated with weekly methotrexate given on a single day in three doses (3×5 mg) than when the same weekly dose was administered equally over six days (6×2.5 mg) [63]. Likewise, stepwise increases in methotrexate dose in patients with juvenile idiopathic arthritis who were nonresponders to standard low-dose methotrexate did not result in improved clinical outcomes [64].

The safety of LD-aminopterin in canine AD was also examined. In contrast to efficacy, there was no relationship between safety and either dose or schedule of administration. As discussed above, the discordance between efficacy and toxicity in relation to dose supports distinct mechanisms for each, as previously suggested by *in vitro* studies [40]. The incidence of AEs in cohorts treated with LD-aminopterin were similar to one another and to the placebo-treated group. In a previous dose-ranging toxicology study in the canine [12], we determined that 0.2 mg/kg L-aminopterin given once-weekly was the lowest dose that caused the first signs of mild toxicity. Thus, the optimal therapeutic dose identified in this trial establishes a therapeutic index with a 14-fold margin of safety.

Subjects from the blinded trial were also optionally able to continue on LD-aminopterin in an open-label extension lasting up to 104 weeks. Of the 75 subjects enrolled in the blinded trial, 62 (83%) enrolled in the extension. The doses used in the extension were 0.007 mg/kg (19%), 0.014 mg/kg (57%) and 0.021 mg/kg (24%). Including the 12 weeks of treatment in the blinded trial, 40 (65%) and 23 (37%) subjects were treated for more than 57 and 84 weeks, respectively. The drug was well-tolerated during chronic therapy and no adverse event required discontinuation of study drug.

The safety profile of weekly methotrexate in the canine at anti-inflammatory doses is not well defined. Weekly treatment of five dogs with CAD with an oral anti-inflammatory dose of methotrexate (0.2 mg/kg) for four weeks resulted in severe vomiting in one subject and fatal hepatic necrosis in two subjects (personal communication by Dr. Thierry Olivry, North Carolina State University). Pond and Morrow reported a similar case of fatal hepatic necrosis in a dog with osteosarcoma treated with methotrexate at an oral dose of 5 mg/m^2^ (0.25 mg/kg) on the first four days of each week [65]. A four-week toxicology study of LD-aminopterin, L-aminopterin and D-aminopterin in beagle dogs (*N* = 6 per cohort, once-weekly oral gavage of 0.5 mg/kg of each enantiomer or 35-fold the anti-inflammatory dose) found no liver histopathology in any cohort (unpublished data). Although data from controlled studies are needed, these observations suggest methotrexate and LD-aminopterin may have different therapeutic indices in the canine.

Options for systemic treatment of human AD include azathioprine, cyclosporine, and methotrexate [5]. A systematic review and meta-analysis of 15 studies and 602 patients determined that cyclosporine consistently decreased the severity of AD [66]. The pooled mean decrease in disease severity was 22% (95% CI, 8–36%) under low-dose cyclosporine (3 mg/kg), and 40% (95%-CI 29–51%) at dosages ≥4 mg/kg. Although effective, a proportion of patients discontinue cyclosporine because of ineffectiveness or side effects, and long-term use raises concerns of nephrotoxicity [67].

Methotrexate has fewer safety concerns than cyclosporine in humans, and was shown in open-label and randomized controlled trials to be an effective treatment of AD [42], [68]. An open-label study evaluated the efficacy and safety of low-dose methotrexate (7.5 mg/week) and cyclosporine (2.5 mg/kg/day) in the treatment of severe AD, and determined there was no statistically significant difference in disease reduction between treatments [41].

Cyclosporine is FDA approved in the United States and elsewhere in the world for the control of CAD. In the pivotal efficacy field trial, four weeks of daily cyclosporine (5 mg/kg) gave a mean (baseline:endpoint) reduction in CADESI (0–360 scale) and PVAS (0–5 scale) in the intent-to-treat population (*N* = 262) of 31.5 (79.0∶47.5) and 1.36 (3.75∶2.39), respectively [51]. The data from this study show that once-weekly LD-aminopterin (0.014 mg/kg, *N* = 15) resulted in a mean (baseline:endpoint) reduction in CADESI of 53 (159∶107), and a reduction in PVAS of 1.9 (7.4∶5.5). Qualitatively, cyclosporine and LD-aminopterin appear to have a similar effect on CAD disease activity. Any formal comparison would require a well-controlled and properly powered head-to-head study.

LD-aminopterin may thus provide an additional therapeutic option to treat AD, but with a better safety profile than either methotrexate [16], [17], [18], [19], [20], or cyclosporine. The efficacy and safety data for LD-aminopterin from this study go toward supporting the rationale for a human trial and provide insights for optimal antifolate dosing in human AD.

## Supporting Information

Table S1 Clinical laboratory adverse events in the open-label trial segment.
(DOCX)

Dataset S1 GS scores.
(CSV)

Dataset S2 Percent change in baseline GS scores.
(CSV)

Dataset S3 PVAS scores.
(CSV)

Dataset S4 CADESI scores.
(CSV)
