# Supplementary material for: LD-Aminopterin in the Canine Homologue of Human Atopic Dermatitis: A Randomized, Controlled Trial Reveals Dosing Factors Affecting Optimal Therapy
Source: PLoS One. 2014 Sep 25;9(9):e108303. doi: 10.1371/journal.pone.0108303 (PMC4177985; doi:10.1371/journal.pone.0108303)
Supplement: Table S1 — Clinical laboratory adverse events in the open-label trial segment. (DOCX) [file pone.0108303.s001.docx]

**Table S1.** Clinical laboratory adverse events in the open-label trial segment.^a^

|  | **LD-Aminopterin** | | |
| --- | --- | --- | --- |
|  | **0.007x1**  **mg/kg** | **0.014x1**  **mg/kg** | **0.021x1**  **mg/kg** |
| **Laboratory Abnormality** | ***N*=12** | ***N*=35** | ***N*=15** |
| Hematocrit Decreased | 0 (0) | 1 (1.6) | 0 (0) |
| Thrombocytosis | 3 (4.8) | 8 (12.9) | 2 (3.2) |
| Leukopenia | 1 (1.6) | 3 (4.8) | 1 (1.6) |
| Lymphopenia | 1 (1.6) | 9 (14.5) | 1 (1.6) |
| Neutropenia | 1(1.6) | 1 (1.6) | 1 (1.6) |
| Eosinophilia | 0 (0) | 1 (1.6) | 1 (1.6) |
| BUN Increased | 2 (3.2) | 4 (6.5) | 0 (0) |
| Creatinine Increased | 0 () | 4 (6.5) | 1(1.6) |
| Alkaline Phosphatase Increased | 2 (3.2) | 16 (25.8) | 6 (9.6) |
| ALT Increased | 1 (1.6) | 7 (11.3) | 1 (1.6) |
| Serum Total Protein Decreased | 0 (0) | 1 (1.6) | 0 (0) |
| **Total** | **11 (17.7)** | **55 (88.7)** | **14 (32.0)** |

^a^ Expressed as *N* and percent of 62 total subjects.
